# Predicting sulfotyrosine sites using the random forest algorithm with significantly improved prediction accuracy

**DOI:** 10.1186/1471-2105-10-361

**Published:** 2009-10-29

**Authors:** Zheng Rong Yang

**Affiliations:** 1 School of Biosciences, University of Exeter, UK Exeter EX4 5DE, UK

## Abstract

**Background:**

Tyrosine sulfation is one of the most important posttranslational modifications. Due to its relevance to various disease developments, tyrosine sulfation has become the target for drug design. In order to facilitate efficient drug design, accurate prediction of sulfotyrosine sites is desirable. A predictor published seven years ago has been very successful with claimed prediction accuracy of 98%. However, it has a particularly low sensitivity when predicting sulfotyrosine sites in some newly sequenced proteins.

**Results:**

A new approach has been developed for predicting sulfotyrosine sites using the random forest algorithm after a careful evaluation of seven machine learning algorithms. Peptides are formed by consecutive residues symmetrically flanking tyrosine sites. They are then encoded using an amino acid hydrophobicity scale. This new approach has increased the sensitivity by 22%, the specificity by 3%, and the total prediction accuracy by 10% compared with the previous predictor using the same blind data. Meanwhile, both negative and positive predictive powers have been increased by 9%. In addition, the random forest model has an excellent feature for ranking the residues flanking tyrosine sites, hence providing more information for further investigating the tyrosine sulfation mechanism. A web tool has been implemented at  for public use.

**Conclusion:**

The random forest algorithm is able to deliver a better model compared with the Hidden Markov Model, the support vector machine, artificial neural networks, and others for predicting sulfotyrosine sites. The success shows that the random forest algorithm together with an amino acid hydrophobicity scale encoding can be a good candidate for peptide classification.

## Background

Tyrosine sulfation is a posttranslational modification (PTM), which introduces a sulfate group to a tyrosine residue in a protein [1-3]. During the modification process, sulfation is catalysed by tyrosylprotein sulfotransferase [4]. A targeted tyrosine for sulfation is normally required to be exposed on a protein surface [5]. Previous studies have indicated that Sulfation is an important anticipator for extracellular protein-protein interactions [6,7]. Studies have shown that sulfation is related to various diseases when a malfunction of a cellular activity occurs. For instance, sulfotyrosine can alter the affinity in some chemokine receptors leading to a downstream signalling cascade which affects the cells involved in acute and chronic events of cellular immunity [8]. Disease-related alterations at the non-reducing termini of chondroitin and dermatan sulfate have been found useful for monitoring proteoglycan metabolism [9]. In biochemistry, sulfation has been recognised as an important contributor to detoxication for endogenous compounds [10]. Sulfation activity has been investigated in various cancer studies such as breast cancer [11-13], lung cancer [14], prostate cancer [15,16], and pancreatic cancer [17-19]. Because of the relevance to various disease, tyrosine sulfation has been the target for drug design for over a decade [20-25].

*In silico *prediction of posttranslational modification sites is a significant activity in bioinformatics. For instance, in ExPASy  various PTM site predictors have been implemented. Specifically, a predictor named as Sulfinator  for sulfotyrosine site prediction has been successfully implemented using Hidden Markov Models (HMM) [26]. The predictor was able to obtain a sensitivity (the accuracy of predicting true sulfotyrosine sites) of 98% and total prediction accuracy of 98%. When the predictor is used on newly sequenced proteins, it is found that the predictor has a particularly low sensitivity although the specificity (the accuracy of predicting unconfirmed sulfotyrosine sites) is high. In this study, a new approach is therefore developed aiming to improve the sensitivity while maintaining the specificity. There is another predictor developed only for tyrosine sulfation sites in animal viruses using Position-Specific-Scoring-Matrix (PSSM) [27]. This approach is very similar to the so-called *h*-function proposed by Poorman [28] 18 years ago. Because only positive peptides are used for scoring, such an approach suffers low specificity when used for making prediction on unseen data [29]. 69 Jackknife simulations were conducted for only positive data. Although it claimed prediction accuracy of 96.43%, the model was actually trained with a carefully selected threshold. The claimed accuracy was observed after tuning the threshold, which is therefore likely over-estimated. Meanwhile, there is no public available tool for the comparison.

In a review paper, some most common features describing the patterns of the residues flanking a tyrosine sulfation site were given [30]. The patterns are found from the residues which flank the experimentally verified tyrosine sulfation sites using a regular expression pattern match approach. This is commonly used in various posttranslational modification pattern analysis projects. The web tool called WebLogos (or sequence logos) is such an application [31]. The reviewer discussed some motif patterns summarised from an earlier study, for instance, Glu and Asp commonly occur between -2 and 2 of a tyrosine sulfation site. However, the regular expression approach suffers two theoretical limitations. First, such an approach assumes that motif positions are mutually independent with a uniform background distribution which may not be true in most applications [32]. Second, the motifs generated this way are sensitive to experimental errors [33]. Machine learning models, on the other had, are more error-tolerate and have been recognised being capable of generalising well on unseen data.

In the common practice of peptide classification, the input for site prediction is normally a symmetrical peptide of consecutive amino acid resides that flank the potentially modified tyrosine. In this study three peptide sizes have been evaluated and the amino acids have been encoded using a hydrophobicity scale [34]. The encoded numerical data of peptides are then treated as inputs for building prediction models using various machine learning algorithms.

The reason of using a hydrophobicity scale is due to its traditional role in analysing the impact of amino acid hydrophobicity on protein structure and potential sites for protein-protein interactions [35]. Hydrophobic amino acids are generally located in the protein interior whereas hydrophilic amino acids are generally located on the protein surface as targets for binding with other molecules. A protein whose surface is composed of mainly negatively charged amino acids such as glutamate and aspartate will bind to a protein with mainly positively-charged molecules such as lysine and arginine [36-40]. This means that the hydrophobicity scale can be one candidate for encoding amino acids for constructing a predictive model. This study has used the Cornette scale [34].

## Results

There are 18 experimentally verified sulfotyrosine sites and 33 unconfirmed sulfotyrosine sites in 15 blind test sequences. Two inferred sulfotyrosine sites were not used for the evaluation. Table 1 shows the prediction result for these sequences using the Sulfinator. In the table, "Actual" means the experimentally verified sulfotyrosine sites while "Predicted" means the predicted sulfotyrosine sites. "Accession" is the accession number from NCBI database. Numbers in bold face are the sulfotyrosine sites missed from the Sulfinator. Numbers in italic are the false sulfotyrosine sites. The total prediction accuracy is 82% with a specificity of 94% and a sensitivity of 61%. The sensitivity is 33% lower than the specificity.

**Table 1:** The prediction result for the 15 blind test sequences using the Sulfinator.

**Accession**	**Actual sites**		**Predicted sites**		
Q9PU41	112		112		
P61073	21		21		
Q9NZ53	97	**118**	97		
A2ZBG5	**110**	**112**			
A2YFB4	80	**82**	80		
Q7 M3V5	114		114	*102*	*131*
P84900	**62**				
Q0VTT9	**62**				
P0C1V8	16		16		
P0C1V7	16		16		
Q800F1	**62**				
P68116	5		5		
P68124	4		4		
P68121	3		3		
P68119	6		6		

Table 2 shows the prediction performances of all machine learning models constructed in this study. In the Table, the figures in bold face represent the models that outperform the Sulfinator. 10, 20, and 30 represent the peptide sizes. "n.a." represents "not available" because the kNN models have no AUR. The models built using the LDA, QDA, CART, 1NN and ANN5 approaches are not compatible with the Sulfinator. Other approaches generate at least one model as accurate as the Sulfinator depending on peptide sizes. All three RF models outperform the Sulfinator, so do all three 5NN models. Four models have achieved >90% total prediction accuracy with improved specificity and sensitivity. The RF models increase the sensitivity by 22%, the specificity by 3%, and the total prediction accuracy by 10% compared with the Sulfinator. The 20-mer SVM model increases the specificity by 6%, the sensitivity by 11%, and the total prediction accuracy by 8%.

**Table 2:** The prediction performances of all machine learning models.

	**10**				**20**				**30**			
	**Spe**	**Sen**	**Tot**	**AUR**	**Spe**	**Sen**	**Tot**	**AUR**	**Spe**	**Sen**	**Tot**	**AUR**

LDA	70	78	73	0.80	76	89	80	0.87	82	83	82	0.88
QDA	85	50	73	0.82	88	44	73	0.80	91	72	84	0.84
CART	91	72	84	n.a.	76	83	78	n.a.	88	83	86	n.a.
1NN	91	72	84	n.a.	88	72	82	n.a.	85	72	80	n.a.
3NN	85	78	82	n.a.	**94**	**72**	**86**	n.a.	**94**	**72**	**86**	n.a.
5NN	**94**	**72**	**86**	n.a.	**97**	**72**	**88**	n.a.	**97**	**72**	**88**	n.a.
7NN	88	67	78	n.a.	88	78	84	n.a.	**94**	**78**	**88**	n.a.
9NN	94	50	78	n.a.	88	78	86	n.a.	**94**	**78**	**88**	n.a.
RF	**97**	**83**	**92**	**0.93**	**97**	**83**	**92**	**0.95**	**97**	**83**	**92**	**0.94**
ANN5	94	28	71	0.81	88	67	80	0.86	88	78	84	0.92
ANN10	100	33	76	0.82	**94**	**78**	**88**	**0.94**	91	72	84	0.92
ANN15	91	56	78	0.86	**97**	**67**	**86**	**0.89**	**94**	**78**	**88**	**0.93**
ANN20	91	56	78	0.88	**94**	**72**	**86**	**0.96**	**94**	**78**	**88**	**0.93**
SVM	87	78	83	0.89	**100**	**72**	**90**	**0.94**	**94**	**78**	**88**	**0.92**

The Chi-square test which has been used in bioinformatics [41] is used to evaluate the significance of the improvement of the **sensitivity**. The test value is 7.93 (p < 0.01). This represents that the new predictor is able to increase the sensitivity significantly compared with the previous one.

**Figure 1:**
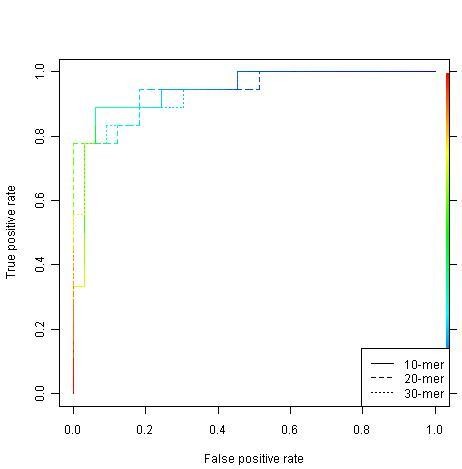
**RF ROC curves for the 10-mer, 20-mer and 30-mer data sets**. The horizontal axes are the false alarm rates (1 - specificity) and vertical axes are the sensitivity. For specific threshold for discriminating between positive (true sulfotyrosine sites) and negative (unconfirmed sulfotyrosine sites) data points, there will be a pair of these two values, i.e., 1 - specificity and sensitivity. A pair of values is then represented by a point in this two-dimensional space. Each curve is made by connecting all these points. A model is said to be robust whether its ROC curve is close to the top left corner. The area under a ROC curve is a quantitative indicator of this robustness.

**Figure 2:**
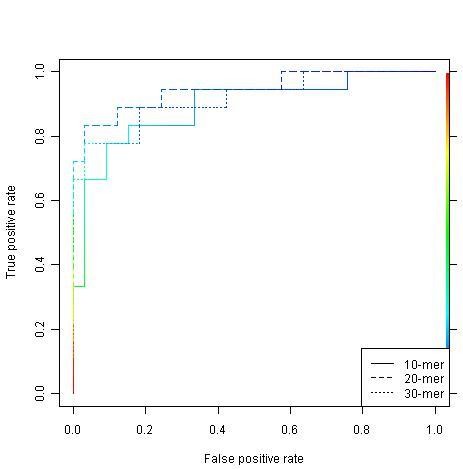
**SVM ROC curves for the 10-mer, 20-mer and 30-mer data sets**. The horizontal axes are the false alarm rates (1 - specificity) and vertical axes are the sensitivity. For specific threshold for discriminating between positive (true sulfotyrosine sites) and negative (unconfirmed sulfotyrosine sites) data points, there will be a pair of these two values, i.e., 1 - specificity and sensitivity. A pair of values is then represented by a point in this two-dimensional space. Each curve is made by connecting all these points. A model is said to be robust whether its ROC curve is close to the top left corner. The area under a ROC curve is a quantitative indicator of this robustness.

Figures 1 and 2 show the ROC curves of the RF and SVM models, respectively. It can be seen that three RF models are consistent while the SVM model built on the 20-mer peptide outperforms the other two SVM models, i.e. the curve is more close to the top-left corner.

## Discussion

**Figure 3:**
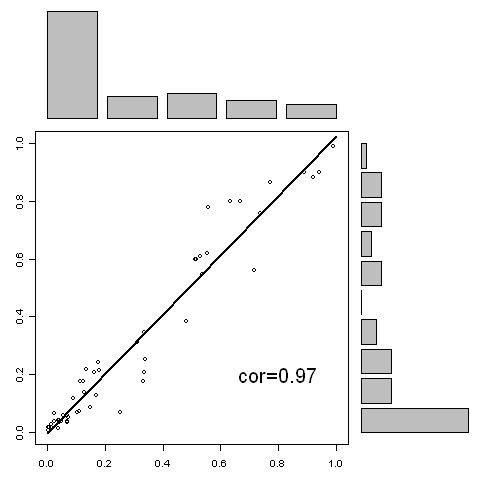
**The correlation of the predictions between 10-mer model predictions (horizontal axis) and the 20-mer model predictions (vertical axis) for the blind data set**.

**Figure 4:**
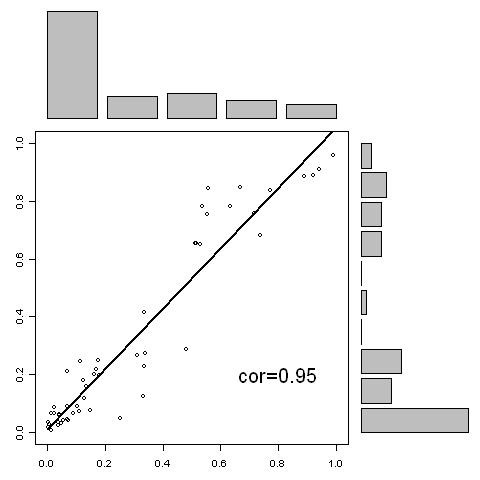
**The correlation of the predictions between 10-mer model predictions (horizontal axis) and the 30-mer model predictions (vertical axis) for the blind data set**.

**Figure 5:**
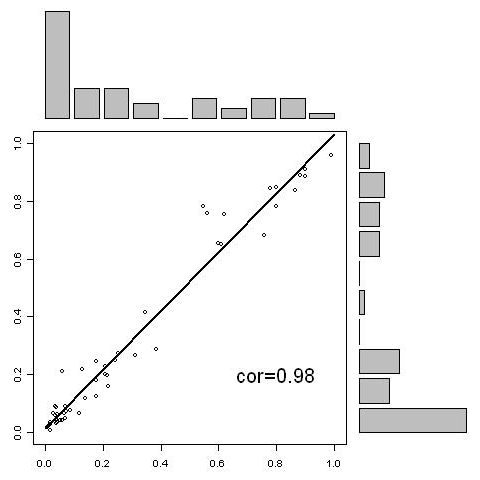
**The correlation of the predictions between 20-mer model predictions (horizontal axis) and the 30-mer model predictions (vertical axis) for the blind data set**.

In order to investigate the consistency among the RF models, correlation analysis is conducted. Figure 3 shows the correlation analysis of the predictions generated from the 10-mer RF model and 20-mer RF model. The correlation is 0.97. Figure 4 shows the correlation analysis of the predictions generated from the 10-mer RF model and 30-mer RF model. The correlation is 0.95. Figure 5 shows the correlation analysis of the predictions generated from the 20-mer RF model and 30-mer RF model. The correlation is 0.98. The high correlation indicates that three RF models are very consistent in prediction demonstrating a high robustness of the algorithm.

**Figure 6:**
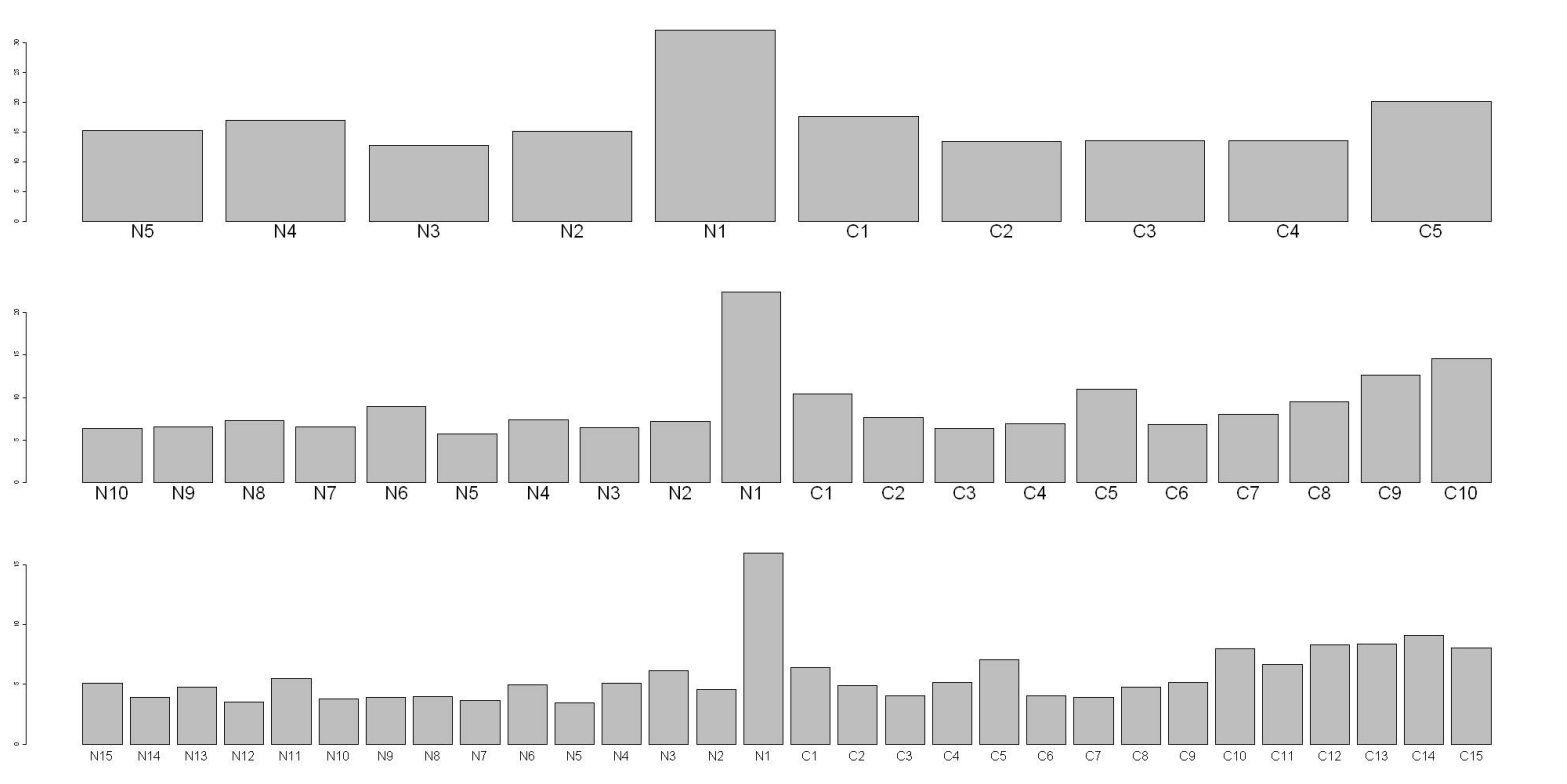
**The ranking results of residues in three RF models**. The horizontal axis represents residue positions in peptides. The upper panel is for the 10-mer data, hence having residue positions ranging from N_5 _to C_5_. The middle panel is for the 20-mer data, hence 20 bars. The lower panel is for the 30-mer data, hence 30 bars. The vertical axis indicates the mean decrease Gini measures.

**Figure 7:**
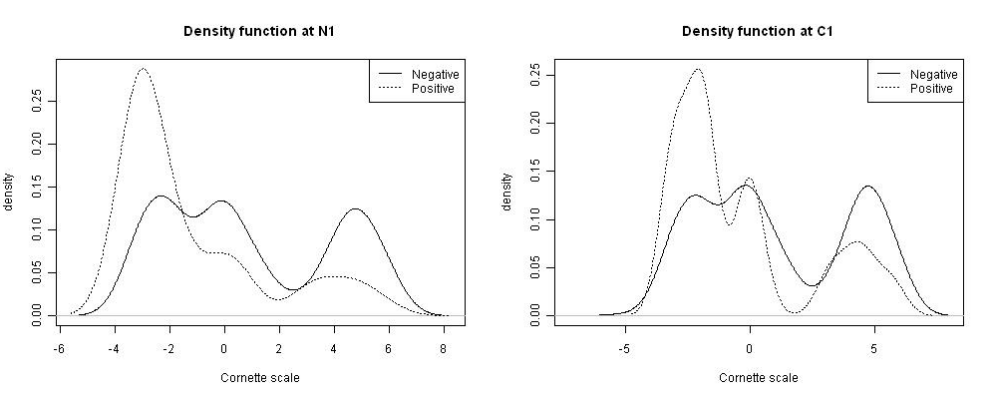
**The conditional density functions drawn at N_1 _and C_1 _residues, respectively**. The horizontal axes represent the Cornette scale values while the vertical axes represent the density values. The density functions are estimated using the kernel approach using the R stats package with default parameter setting. The graph shows that the density functions drawn at N_1 _demonstrate a larger separation between two classes while this difference is getting smaller for the residue C_1_, which does not have a high rank value from RF models. Note that negative means unconfirmed sulfotyrosine whilst positive means experimentally verified sulfotyrosine.

Figure 6 shows the ranking results from three RF models (mean decrease Gini gain [42,43]). It can be seen that residue N_1 _has been consistently highly ranked. Other residues with higher rank values are C_5_, C_10_, and C_14_. Based on the conditional density functions of N_1_and C_1 _shown in Figure 7, it can be seen that residue N_1_does contribute more to the classification of the two classes of peptides compared with C_1_. Because tyrosine sulfation plays a role in protein-protein interaction, several laboratorial works have found that N-terminal residues contribute to sulfotyrosines, i.e., the N-terminal domain of sulfotyrosines involve direct protein-protein interaction through a P-selection [44-48].

The major differences between the Sulfinator and the predictors constructed in this paper are the use of different algorithms as well as the different presentation approaches of amino acids to a machine learning model. HMM does not need an encoding process while the predictors constructed in this study use a hydrophobicity scale to encode the amino acids. It is known that the random forest algorithm and the support vector machine algorithm have been well-known in improving the generalisation capability of a model. The significant improvement in the prediction accuracy in blind data these models can result from the use of RF and SVM algorithms and the use of hydrophobicity scale.

Finally, a single RF predictor is built using whole training data coded using 20-mer peptides (excluding any blind sequences). The RF predictor, a C program which is used to extract 20-mer peptides from a query sequence and encoding peptides using the Cornette scale, the whole training sequences and the blind sequences are available in the web site  for free use, where a web tool is also available.

The RF predictor is then used to make prediction on the 15 blind sequences. Its performance is the same as that obtained from cross-validation models. For instance, protein Q9PU41 is a Cholecystokinin with 130 residues. It is related to the release of pancreatic enzymes in the gut [49]. A high degree of identity was found between the sequence from chicken and chinchilla which inferred a homologous function [50]. The sulfotyrosine is the first residue of the peptide Cholecystokinin-7 (112-118). The residue has been accurately predicted by both cross-validation RF predictions and the single RF predictor, as well as using Sulfinator. Protein A2ZBG5 is a Phytosulfokines 2. Two sulfotyrosine sites have been found in the peptide Phytosulfokine-beta (110-113). This protein is associated with plant cell differentiation, organogenesis, somatic embryogenesis and cell proliferation. The sulfated tyrosine is for binding to a putative membrane receptor [51]. In this case both sites have been accurately predicted using the RF model. However, Sulfinator failed to predict both. The RF predictor failed to predict three sulfotyrosine sites in extracellular proteins (Y21 in P61073 as well as Y97 and Y118 in Q9NZ53) while Sulfinator failed to predict one of these three sites (Y118 in Q9NZ53). Details of the analysis of all the predictions can be seen in Table 3. In the Table, "Site" represents the experimentally verified sulfotyrosine sites as well as those tyrosine residues which are not experimentally verified sulfotyrosine sites. If the value in the "TURE" column is 1, it represents that the site is an experimentally verified one. Sulfinator represents the predictions of the Sulfinator tool, where "Y" represents predicted sulfotyrosine sites including false positives. The sites 102 and 131 of Q7 M3V5 are missed experimentally verified sulfotyrosine sites. Blanks represent predicted non- sulfotyrosine sites. "RF1" represents the prediction (posterior probabilities) of cross-validation predictions while "RF2" represents the predictions (posterior probabilities) made by a single RF predictor. "Peptide" represents the segments in which the sulfotyrosine sites sit. "Region" represents the protein in which the sulfotyrosine sites are.

**Table 3:** The prediction details of 15 blind testing proteins.

**Protein**	**Site**	**TRUE**	**Sulfinator**	**RF1**	**RF2**	**Peptide**	**Region**
Q9PU41	112	1	Y	0.9884	0.988	Cholecystokinin-7	
Q9PU41	2	0		0.1384	0.122		
Q9PU41	79	0		0.0588	0.036		
P61073	21	1	Y	0.2528	0.158		Extracellular
P61073	7	0		0.176	0.092		
P61073	12	0		0.128	0.264		
P61073	45	0		0.0188	0.006		
P61073	65	0		0.086	0.074		
P61073	76	0		0.1776	0.164		
P61073	103	0		0.0084	0.008		
P61073	116	0		0.04	0.036		
P61073	121	0		0.034	0.03		
P61073	135	0		0.0284	0.022		
P61073	157	0		0.0404	0.034		
P61073	184	0		0.0408	0.026		
P61073	190	0		0.0688	0.062		
P61073	219	0		0.016	0.02		
P61073	255	0		0.0152	0.02		
P61073	256	0		0.0388	0.04		
P61073	302	0		0.0176	0.02		
Q9NZ53	97	1	Y	0.0692	0.076		Extracellular
Q9NZ53	118	1		0.2072	0.236		
Q9NZ53	391	0		0.0672	0.06		
Q9NZ53	481	0		0.1168	0.112		
Q9NZ53	498	0		0.1776	0.152		
Q9NZ53	522	0		0.0384	0.052		
A2ZBG5	110	1		0.618	0.606	Phytosulfokine-beta	
A2ZBG5	112	1		0.7988	0.814		
A2YFB4	80	1	Y	0.5476	0.596	Phytosulfokine-beta	
A2YFB4	82	1		0.7788	0.796		
Q7 M3V5	114	1	Y	0.2064	0.226	Callisulfakinin-1	
Q7 M3V5	2	0		0.3116	0.358		
Q7 M3V5	12	0		0.0608	0.064		
Q7 M3V5	56	0		0.0528	0.044		
Q7 M3V5	64	0		0.2184	0.232		
Q7 M3V5	65	0		0.0348	0.038		
Q7 M3V5	81	0		0.3856	0.378		
Q7 M3V5	102	0	Y	0.072	0.054		
Q7 M3V5	131	0	Y	0.5604	0.592		
P84900	62	1		0.608	0.582	Phyllokinin	
Q0VTT9	62	1		0.598	0.57	[Thr6, Val10]-phyllokinin	
P0C1V8	16	1	Y	0.8816	0.92	Alpha-conotoxin AnIC	
P0C1V7	16	1	Y	0.8988	0.93	Alpha-conotoxin AnIA	
Q800F1	62	1		0.5984	0.57	[Thr6]-phyllokinin	
P68116	5	1	Y	0.7984	0.816	Fibrinopeptide B	
P68116	2	0		0.3464	0.338		
P68124	4	1	Y	0.8984	0.902	Fibrinopeptide B	
P68121	3	1	Y	0.7584	0.742	Fibrinopeptide B	
P68119	6	1	Y	0.8648	0.864	Fibrinopeptide B	

It is also important to see how confident we trust the predictions made by a model and whether this new approach is making a significant contribution to prediction accuracy compared with old models. For this we investigate the properties of the negative and positive predictive powers [52-55]. The negative predictive power measures how likely a negative prediction is true. In other words, it measures the probability that a prediction of unconfirmed sulfotyrosine is a true unconfirmed sulfotyrosine. It is calculated by the fraction of correctly identified unconfirmed sulfotyrosine sites over the total predicted unconfirmed sulfotyrosine sites. The positive predictive power then measures the probability that a sultyrosine prediction is a true sulfotyrosine. This is calculated by the fraction of correctly identified true sulfotyrosine sites over the total predicted sulfotyrosine sites. Given the confusion matrix made by testing the blind sequences as in Table 4, we can work out these two measurements. In the Table, "Negative" represents unconfirmed sulfotyrosine and "Positive" represents experimentally verified sulfotyrosine. The left panel is the result obtained when using Sulfinator while the right panel is the result generated by the RF model based on the 20-mer data. When using Sulfinator, the negative and the positive predictive powers are 82% and 85%, respectively. However they are 91% and 94%, respectively, when using the RF models. It can be seen that the confidence of trusting an unconfirmed sulfotyrosine site has increased by 9% using the RF models and the confidence of trusting a sulfotyrosine site has improved by 9% as well.

**Table 4:** Confusion matrices for Sulfinator and the RF models in this study.

	**Sulfinator**			**RF model**		
	**Negative**	**Positive**		**Negative**	**Positive**	
Negative	31	2	94%	32	1	97%
Positive	7	11	61%	3	15	83%
	82%	85%	82%	91%	94%	92%

## Conclusion

This paper has presented a new predictor for sulfotyrosine sites in protein sequences. The sequences annotated after 2002 are used as the blind test data for comparing the models constructed using various other machine learning algorithms in this study and Sulfinator, a prediction tool established in 2002. Through evaluation, it has been found that the predictors constructed using the random forest algorithm and the support vector machine algorithm show significantly improved prediction accuracy compared with Sulfinator. The random forest models demonstrate consistently better performance. Using the RF models, the sensitivity is increased by 22%, the specificity is increased by 3%, and the total prediction accuracy is increased by 10% compared with the sulfinator. Both negative and positive predictive powers have been increased by 9% using the RF models. The 20-mer RF model is the method of choice for implementing a predictor because it has the highest AUR.

## Methods

### Data

363 proteins with experimentally verified sulfotyrosine sites were collected from NCBI [56]. Two rules were used for pre-processing the data. First, any sequence without an experimentally verified sulfotyrosine was removed. Second, the CD-HIT algorithm [57-59] was used to remove sequences with ≥ 90% similarity. Applying these two rules gave 94 sequences for the study. Among them, 79 were annotated before 2002 (inclusive) while the rest 15 were annotated after 2002. 79 early annotated sequences were used to train and select a predictor while 15 later annotated sequences were used as blind test data set for the comparison with the Sulfinator. The separation of data in 2002 was because the Sulfinator was developed in 2002. All the sequences annotated after 2002 should therefore be blind to the predictor.

### Peptide formation and coding

All the tyrosines were extracted from the 79 test sequences. Following a common procedure in constructing a PTM site predictor, a peptide was formed symmetrically using both N-terminal and C-terminal consecutive residues flanking a tyrosine. It was denoted by N_m_-N_m-1_-...N_1_- C_1_-...C_m-1_-C_m_. Here, 2 m was the number of flanking residues in a peptide with N-_k _as an N-terminal residue and C-_k _as a C-terminal residue of a tyrosine. Note that the tyrosine in the middle was not used because it was identical in all peptides. Three peptide sizes were used, i.e. 2 m = 10, 20, and 30. A peptide generated from an experimentally verified sulfotyrosine was labelled as positive while a peptide generated from an unconfirmed sulfated tyrosine (has not yet been declared as a sulfated tyrosine) was labelled as negative. 132 positive and 626 negative non-repeated peptides were found in 79 sequences. Here, "non-repeated" indicated that any repeated peptide was removed. Each peptide was encoded to a numeric vector using the Cornette hydrophobicity scale.

### Model construction

The machine learning algorithms used in this study were linear discriminant analysis (LDA) [43,60], quadratic discriminant analysis (QDA) [43,60], k-nearest neighbour (kNN) [43,60], classification and regression tree (CART) [61], the random forest algorithm (RF) [62], the support vector machine (SVM) [63] and artificial neural network (ANN) [64]. Because RF is a newly developed machine learning algorithm, a brief description of it is placed below. All these algorithms were available in the R programming environment (built by the R project, ). The hidden neurons of ANN were 5, 10, 15, and 20. The numbers of nearest neighbours were 1, 3, 5, 7, and 9. The distance used in kNN was the Euclidean distance. The radial basis kernel function of the SVM was used with the smoothing parameter as 0.2. The cost parameter of the SVM was 100. The default parameters of LDA, QDA, CART, and RF were used.

### Model evaluation

Models were evaluated by the sensitivity (Sen, the prediction accuracy of true sulfotyrosine sites), the specificity (Spe, the prediction accuracy of unconfirmed sulfotyrosine sites), the total accuracy (Tot), and receiver operating characteristics (ROC) analysis [65]. ROC was used to measure whether a model was robust. The areas under ROC curves (AURs) were used as a quantitative indicator of model robustness. The five-fold cross-validation approach [66] was used for model evaluation. ROC curves were drawn using the ROCR R package [67] and the area under ROC curves was calculated using the caTools R package .

### The random forest algorithm

The random forest algorithm is a newly developed machine learning algorithm [62]. The basic idea is to construct many trees using random vectors sampled from a data set. For the kth tree, a random vector is generated independently from the random vectors generated for the past k-1 trees. The remaining data are used for prediction. The approach of sampling random vectors is similar to bootstrap, i.e. the replacement sampling approach, which has also been applied to analysing biological data [68]. For each node in a tree, a small fraction of variables is randomly selected. The best split for the node is based on the prediction error. Each tree is fully grown without pruning. RF is able to provide a number of excellent features, for instance, the capability of handling a large number of variables, ranking the variables, and detecting the interaction among the variables. The algorithm has been recently applied to various biological data mining projects, for example, the prediction of the interactions between HIV-1 and human proteins using gene expression data [69], the analysis of differential gene expression [70], the diagnosis of ulcerative colitis based on gene expression data [71], the detection of cancers [72], the prediction of childhood leukaemia using gene expression data [73], and the prediction of protein-protein interactions [74]. All these applications show that the random forest algorithm outperforms some other algorithms.

## Authors' contributions

ZRY collected the data, wrote and debugged C and R scripts for model construction and evaluation as well as the presentation of the work, implemented the web tool using JavaScript and Perl, and completed the manuscript preparation.
